# Response of the Anaerobic Methanotrophic Archaeon *Candidatus* “*Methanoperedens nitroreducens*” to the Long-Term Ferrihydrite Amendment

**DOI:** 10.3389/fmicb.2022.799859

**Published:** 2022-04-18

**Authors:** Chen Cai, Gaofeng Ni, Jun Xia, Xueqin Zhang, Yue Zheng, Bingqing He, Esteban Marcellin, Weiwei Li, Jiaoyang Pu, Zhiguo Yuan, Shihu Hu

**Affiliations:** ^1^CAS Key Laboratory of Urban Pollutant Conversion, Department of Environmental Science and Engineering, University of Science and Technology of China, Hefei, China; ^2^Australian Centre for Water and Environmental Biotechnology, The University of Queensland, St Lucia, QLD, Australia; ^3^State Key Laboratory of Marine Environmental Science, College of the Environment and Ecology, Xiamen University, Xiamen, China; ^4^Australian Institute for Bioengineering and Nanotechnology, The University of Queensland, St Lucia, QLD, Australia; ^5^State Key Laboratory of Coal Mine Disaster Dynamics and Control, Chongqing University, Chongqing, China

**Keywords:** anaerobic oxidation of methane, Fe(III) reduction, ANME archaea, extracellular electron transfer, multiheme *c*-type cytochromes, methanogen, acetate

## Abstract

Anaerobic methanotrophic (ANME) archaea can drive anaerobic oxidation of methane (AOM) using solid iron or manganese oxides as the electron acceptors, hypothetically *via* direct extracellular electron transfer (EET). This study investigated the response of *Candidatus* “*Methanoperedens nitroreducens* TS” (type strain), an ANME archaeon previously characterized to perform nitrate-dependent AOM, to an Fe(III)-amended condition over a prolonged period. Simultaneous consumption of methane and production of dissolved Fe(II) were observed for more than 500 days in the presence of *Ca.* “*M. nitroreducens* TS,” indicating that this archaeon can carry out Fe(III)-dependent AOM for a long period. *Ca.* “*M. nitroreducens* TS” possesses multiple multiheme *c*-type cytochromes (MHCs), suggesting that it may have the capability to reduce Fe(III) *via* EET. Intriguingly, most of these MHCs are orthologous to those identified in *Candidatus* “*Methanoperedens ferrireducens*,” an Fe(III)-reducing ANME archaeon. In contrast, the population of *Ca.* “*M. nitroreducens* TS” declined and was eventually replaced by *Ca.* “*M. ferrireducens*,” implying niche differentiation between these two ANME archaea in the environment.

## Introduction

Anaerobic oxidation of methane (AOM) occurs in a wide range of natural and anthropogenic environments and plays a crucial role in mitigating the global emission of methane by converting this greenhouse gas to less potent CO_2_ (Knittel and Boetius, 2009). In marine settings, AOM contributes to consuming >90% of the methane produced *via* methanogenesis, which makes the ocean a minor methane source (Hinrichs and Boetius, 2002; Reeburgh, 2007). AOM also prominently controls methane flux in some non-marine environments (Hu et al., 2014; Segarra et al., 2015). It is estimated that AOM reduces over 50% of methane emissions in freshwater wetlands, the largest natural methane source (Segarra et al., 2015).

It has been well-documented that AOM can be coupled to the reduction of sulfate, nitrate, and nitrite (Welte et al., 2016; Bhattarai et al., 2019). These AOM processes are conducted by either anaerobic methanotrophic (ANME) archaea or NC10 bacteria (Knittel and Boetius, 2009; Ettwig et al., 2010). Theoretically, AOM coupling to Fe(III) reduction is also thermodynamically favorable. A number of recent incubation studies, in fact, have shown that some ANME species belonging to ANME-2 cluster are able to conduct Fe(III)-dependent AOM (Ettwig et al., 2016; Scheller et al., 2016; Cai et al., 2018; Li et al., 2021). *Candidatus* “*Methanoperedens ferrireducens*,” affiliated with family *Candidatus* “Methanoperedenaceae” (formerly ANME-2d), could use ferrihydrite as the electron acceptor in a long-term incubation (Cai et al., 2018). Intriguingly, a few other ANME-2 archaea using electron acceptors such as sulfate and nitrate also showed the capability to reduce Fe(III) (Ettwig et al., 2016; Scheller et al., 2016). For instance, *Candidatus* “*Methanoperedens nitroreducens* TS” (type strain) has been characterized to grow on nitrate (Haroon et al., 2013), while a *Ca.* “*M. nitroreducens*”-like archaeon (strain MPEBLZ) could perform Fe(III)-dependent AOM in the absence of nitrate (Ettwig et al., 2016). In addition, marine ANME-2 archaea normally mediate sulfate-dependent AOM in cooperation with sulfate-reducing bacteria (Knittel and Boetius, 2009). Using Fe(III) in substitution for sulfate, the ANME-2 archaea reduced Fe(III) without syntrophic interaction with their bacterial partners (Scheller et al., 2016).

It is hypothesized that ANME archaea catalyze Fe(III) reduction *via* extracellular electron transfer (EET; McGlynn et al., 2015; Ettwig et al., 2016; Scheller et al., 2016; Cai et al., 2018; Leu et al., 2020). In dissimilatory metal-reducing microorganisms such as *Geobacter* and *Shewanella*, EET is carried out *via* multiheme *c*-type cytochromes (MHCs; Shi et al., 2016). Likewise, metagenomic analyses revealed that ANME archaea harbor genes encoding numerous MHCs (Meyerdierks et al., 2010; Wang et al., 2014; McGlynn et al., 2015). The number and size of the MHCs in these ANME archaea are comparable to, or even larger than, those in metal-reducing bacteria (Shi et al., 2007; McGlynn et al., 2015; Cai et al., 2018; Leu et al., 2020). For instance, ANME-1 and ANME-2a archaea encode 11 (Meyerdierks et al., 2010) and 16 species (Wang et al., 2014; McGlynn et al., 2015) of MHCs, respectively. Members of *Ca.* “Methanoperedenaceae” encode larger numbers of MHCs (25–46 species) (Haroon et al., 2013; Ettwig et al., 2016; Cai et al., 2018; Leu et al., 2020). Intriguingly, some of the ANME species encode MHCs with a large number of hemes (e.g., a 113-heme MHC in *Candidatus* “*Methanoperedens manganireducens*”) (Leu et al., 2020). Moreover, MHCs showed high expression levels concomitant with metal reduction by ANME archaea (Cai et al., 2018; Leu et al., 2020). Together, these results strongly suggest that MHCs of ANME archaea are involved in EET, which allows these microorganisms to respire metal oxides such as Fe(III).

Increasing geochemical studies have provided evidence that AOM can be driven by Fe(III) reduction in many aquatic environments (Beal et al., 2009; Crowe et al., 2011; Sivan et al., 2011; Amos et al., 2012; Wankel et al., 2012; Segarra et al., 2013; Riedinger et al., 2014; Egger et al., 2015; Egger et al., 2016). In fact, iron oxides are prevalent in freshwater bodies (Martin and Meybeck, 1979) and soils (Deshpande et al., 1964), and large amounts of iron (∼730 Tg/year) are further transported from continents to oceans *via* rivers annually (Martin and Meybeck, 1979). These environments are also characterized as important methane sources and/or sinks (Topp and Pattey, 1997; Conrad, 2009; Knittel and Boetius, 2009). Thus, it can be predicted that Fe(III)-dependent AOM takes place in a variety of environments rich in iron and methane, making this bioprocess a methane sink with potential global importance.

For in-depth understanding of Fe(III)-dependent AOM in nature, it is critical to identify the indigenous microorganisms responsible for this process. A recent study, in fact, has reported that *Ca.* “*M. nitroreducens*”-like archaea exhibited AOM activity in an iron-rich, low-sulfate freshwater lake sediment (Weber et al., 2017). Furthermore, as aforementioned, Fe(III) reduction by *Ca.* “*M. nitroreducens* MPEBLZ” has been demonstrated (Ettwig et al., 2016). Thus, it is likely that *Ca.* “*M. nitroreducens* TS” or *Ca.* “*M. nitroreducens* MPEBLZ” are potential candidates for conducting Fe(III)-dependent AOM in the environment. However, evidence for these archaea to grow on Fe(III) condition is currently lacking (In ‘t Zandt et al., 2018).

The aim of this study was to investigate whether *Ca.* “*M. nitroreducens*” can adapt to a Fe(III)-amended condition over the long-term periods. An inoculum dominated by *Ca.* “*M. nitroreducens* TS” was seeded in a bioreactor amended with an environmentally relevant form of Fe(III) oxide (ferrihydrite) and incubated for 800 days. Notably, *Ca.* “*M. ferrireducens*” has been indicated to be an obligate methane-dependent Fe(III) reducer as it lacks nitrate reductase when compared to *Ca.* “*M. nitroreducens*” (Cai et al., 2018). Therefore, to facilitate an evaluation of the response of *Ca.* “*M. nitroreducens*” to the Fe(III) condition, the incubation conditions for *Ca.* “*M. ferrireducens*” were employed in this study (Cai et al., 2018). Bioreactor performance and microbial community were monitored throughout the incubation. Metagenomic analysis was performed to reveal the metabolic capacity of *Ca.* “*M. nitroreducens* TS” and other key microorganisms.

## Materials and Methods

### Biomass Source

Biomass was originally from a culture dominated by *Ca.* “*M. nitroreducens* TS” (Haroon et al., 2013). In this study, the biomass was taken from a parent bioreactor performing nitrate-dependent AOM and anammox. The total volume of the parent bioreactor was 5.6 L, consisting of 4.6 L mixed biomass and 1.0 L headspace. The parent bioreactor was supplied with methane, nitrate, and ammonium and operated at 24 ± 1°C and neutral pH (7.0–7.5). At the time of biomass sampling for both short-term and long-term tests, the AOM rate of the parent bioreactor was 301.1 μM day^–1^, along with a nitrate reduction rate of 1,130.6 μM day^–1^. It indicated that AOM was predominantly coupled to nitrate reduction, which was in line with the abundance of *Ca.* “*M. nitroreducens* TS” (∼30%) in the microbial community. The nitrite-reducing methanotrophic bacterium (*Candidatus “Methylomirabilis oxyfera*”) only accounted for less than 1%, implying its contribution to methane oxidation was minimal. Ammonium was consumed by anammox bacterium (*Candidatus “Kuenenia stuttgartiensis*”) at a rate of 881.5 μM day^–1^, which accounted for the complete removal of nitrite produced by *Ca.* “*M. nitroreducens* TS”.

### Short-Term Batch Tests

Metagenomic analysis showed that *Ca.* “*M. nitroreducens* TS” only shares an average nucleotide identity (ANI) of 77.4% with *Ca.* “*M. nitroreducens* MPEBLZ” (Supplementary Figure 3) (Arshad et al., 2015), which was demonstrated to perform Fe(III)-dependent AOM (Ettwig et al., 2016). Thus, it necessitates an assessment of the capability of *Ca.* “*M. nitroreducens* TS” to carry out this process. One 230 ml batch reactor (A1) was flushed with N_2_ to remove oxygen. A1 was then inoculated with 100 ml biomass. Methane was supplied by sparging gas mix (90% CH_4_, 5% CO_2_, and 5% N_2_) through the liquid phase for 3 min. The batch tests were conducted in two stages. In Stage I, residual nitrate in the inoculum was served as the electron acceptor for AOM. In Stage II, after nitrate was depleted, Fe(III) citrate (Sigma-Aldrich, United States) was added, as the sole electron acceptor, at a concentration of 4 mM. Another batch reactor (A2) was set up and operated identically to A1 as a control. However, sodium citrate (Sigma-Aldrich, United States), instead of Fe(III) citrate, was supplied to A2 in Stage II. Gas samples were taken every 1–3 days for methane and N_2_ measurement. AOM rate equals the methane consumption rate, which was determined from the measured methane concentration through linear regression (Haroon et al., 2013). Nitrate was monitored using the test strips (Merck, Germany) until it was depleted in Stage I.

### Incubation Under the Long-Term Ferrihydrite Amendment

To evaluate the response of *Ca.* “*M. nitroreducens* TS” to the Fe(III) condition in a long-term period, a bioreactor was inoculated with biomass and supplied with Fe(III) in the form of ferrihydrite as the sole electron acceptor. In total, 300 ml biomass was mixed with 600 ml medium in a 1.1 L bioreactor using a magnetic stirrer at 300 rpm. The bioreactor was operated at 24 ± 1°C and neutral pH.

The entire incubation period was divided into two stages. In Stage I (Days 0–47), the bioreactor was supplied with nitrate as the electron acceptor for AOM. Methane was replenished by flushing the bioreactor with a gas mix (90% CH_4_, 5% CO_2_, and 5% N_2_) every 1–2 weeks. Gas samples were taken daily for methane measurement, and liquid samples were taken two times each week for nitrate, nitrite, and ammonium measurement. AOM rate was determined in an interval of 1 week. The average AOM rate of Stage I was calculated based on the AOM rate determined each week. A biomass sample was taken at the end of this stage for 16S rRNA gene sequencing. In Stage II (Days 48–800), ferrihydrite was periodically added as the sole electron acceptor (Figure 2). Every 3 months, the stirrer was stopped to settle biomass overnight, and 10% of the supernatant (∼90 ml, negligible biomass) was replaced with fresh medium. As AOM rate dramatically decreased in this stage, methane was supplied less frequently (every 1–2 months). Gas samples were taken every 3–5 days for methane measurement. Liquid samples were taken every 1–2 weeks for dissolved Fe(II), nitrate, nitrite, ammonium, and acetate measurement. Biomass samples were taken every 1–3 months for 16S rRNA gene sequencing to monitor the shift of the microbial community.

**FIGURE 1:**
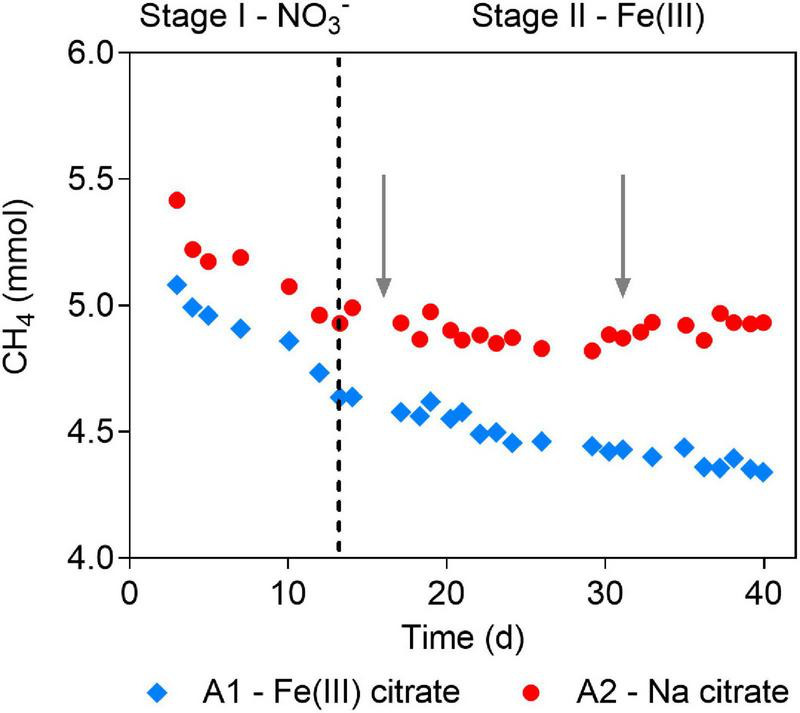
Methane profiles before and after Fe(III) addition in the short-term batch tests. Stages are divided by dashed line. In Stage I, nitrate was the electron acceptor for anaerobic oxidation of methane (AOM) in both A1 and A2. In Stage II, Fe(III) citrate was added to A1 as the electron acceptor for AOM, and sodium citrate was added to A2. Gray arrows show the addition of Fe(III)- and sodium citrate to A1 and A2, respectively.

**FIGURE 2:**
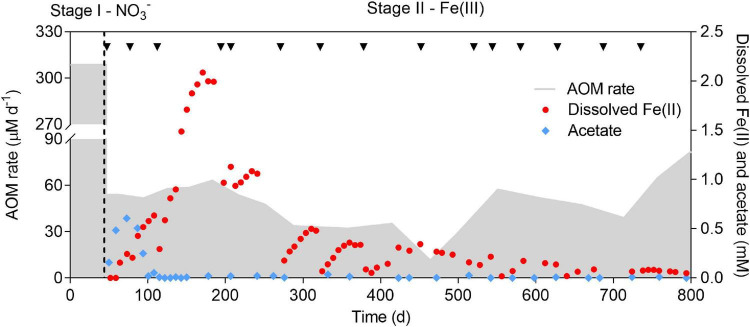
Profiles of AOM rate, dissolved Fe(II), and acetate concentration over a period of 800 days. The incubation period was divided into two stages (indicated by the dashed line). Nitrate and ferrihydrite were supplied as the electron acceptors in Stage I and II, respectively. Black arrows show the addition of ferrihydrite. The sharp drops of dissolved Fe(II) were due to abiotic adsorption by ferrihydrite. Medium (∼10%) was replaced eevery 3 months in Stage II.

### Chemical Analyses

Gaseous methane was measured using a gas chromatograph (GC) (Agilent 7890A, United States) equipped with a HayeSep Q column (mesh size 80/100) (Restek, United States). Dissolved Fe(II) was measured using an Inductively Coupled Plasma-Optical Emission Spectrophotometer (ICP-OES) (PerkinElmer Optima 7300DV, United States). Nitrate, ammonium, and nitrite were measured using a Flow Injection Analyzer (FIA) (Lachat QuickChem8000, United States). Acetate was measured using a GC equipped with a flame ionization detector (PerkinElmer, United States).

### DNA Extraction

For DNA extraction, a 0.4 ml biomass sample was used. DNA was isolated using FastDNA Spin Kit for Soil (MP Bio, United States) according to the manufacturer’s protocol. The concentration and quality of the extracted DNA were assessed using a Nanodrop spectrophotometer (Thermo Fisher Scientific, United States).

### 16S rRNA Gene Amplicon Sequencing and Quantitative PCR

The extracted DNA was delivered to the Australian Centre for Ecogenomics (ACE) at The University of Queensland for 16S amplicon sequencing. In addition, the abundance of *Ca.* “*M. nitroreducens* TS,” methanogens, total bacteria, and total archaea were determined through quantitative PCR (qPCR). The qPCR results were further used for kinetic evaluation of the decay rate of *Ca.* “*M. nitroreducens* TS”. The details can be found in the Supplementary Material.

### Taxonomic Placement, Functional Annotation, and the Construction of the Phylogenomic Tree

DNA samples taken on days 168 and 571 were also used for metagenomic analysis. Library preparation, metagenomic sequencing, quality control, assembly, and binning were described in the Supplementary Material. Phylogeny-based taxonomy was inferred using GTDB-tk version 0.3.2 (with database version r89) (Parks et al., 2018; Chaumeil et al., 2019). The similarity of ANME-associated MAGs with known representatives within the genus *Ca*. “Methanoperedens” was assessed using fastANI version 1.3 (Jain et al., 2018) by calculating pair-wise ANI together with 8 whole-genome sequences from *Ca*. “Methanoperedens” downloaded from the NCBI Refseq (Pruitt et al., 2007) and Genbank (Benson et al., 2010) databases. Open reading frames (ORFs) were called and annotated using Prokka 1.14.0 (Seemann, 2014). Orthology prediction and additional annotation were carried out using eggnog-mapper version 2.0.0 (database version 5.0) (Huerta-Cepas et al., 2017) against Gene Ontology (GO; Consortium, 2004), Kyoto Encyclopedia of Genes and Genomes (KEGG; Kanehisa and Goto, 2000), and clusters of orthologous groups (COG; Koonin, 2002) databases.

A maximum-likelihood phylogenomic tree was constructed using the proteomes of three archaeal MAGs associated with ANME archaea or methanogens together with proteomes of 15 representatives from the genera *Ca*. “Methanoperedens” and *Methanosarcina* were obtained from the NCBI public repository. This was achieved by concatenating 400 universal marker genes in Phylophlan3 (Segata et al., 2013), followed by multiple sequence alignment in muscle version 3.8.31 (Edgar, 2004) and tree inference in IQ-TREE version 2.0, with 1,000 bootstrap iterations (Nguyen et al., 2014). The visualization and manual curation of the phylogenomic tree were carried out in interactive tree of life (ITOL; Letunic and Bork, 2006) and Adobe Illustrator.

### Identification and Orthology Inference of Multiheme *c*-Type Cytochromes

*C*-type cytochromes were first identified from the archaeal proteomes using the “hmmsearch” function of HMMER (version 3.2.1) with the multiheme cytochrome superfamily HMM models (Johnson et al., 2010). They were later confirmed as cytochrome *c* using the BLASTP (Altschul et al., 1997) web service against the NCBI non-redundant (nr) database (updated 05 June 2020). Next, the amount of CXXCH motifs within these cytochromes was determined using the ExPASy web service (Artimo et al., 2012) to search against the PROSITE database (Hulo et al., 2006), and cytochromes containing ≥3 CXXCH motifs were retained as MHCs. Afterwards, the cellular allocation of the MHCs was predicted using the SignalP 5.0 (Almagro Armenteros et al., 2019) by identifying signal peptides (SPs) found in transmembrane proteins. Only MHCs with SPs were considered relevant to extracellular electron transport (Leu et al., 2020). To avoid false detection of orthologous genes by naive similarity score-based approaches, orthologous MHCs were identified in three steps by first inferring orthogroups from protein sequences, which were used to infer gene trees, and the gene trees were subsequently used to identify orthologous MHCs. This was done in Orthofinder (version 2.3.12) (Emms and Kelly, 2019).

### Assessment of Acetate Metabolism

Four genes are predicted to be critical in acetate consumption, namely, *acd*, encoding acetyl-CoA synthetase (ADP-forming); *acs*, encoding acetyl-CoA synthetase (AMP-forming); *ack*, encoding acetate kinase; and *pta*, encoding phosphotransacetylase (Brown et al., 1977; Bräsen and Schönheit, 2001; Wolfe, 2005). Public proteins of these enzymes were downloaded from the Uniprot database (accessed on 22 February 2020) (The UniProt Consortium, 2017) and were used to create reference packages in GraftM version 0.13.1 (Boyd et al., 2018). Later, homologs of *acd*, *acs*, *ack*, and *pta* in the MAGs were revealed using the GraftM “graft” function.

## Results

### Short-Term Performance of the Culture Using Fe(III) as the Electron Acceptor

In Stage I of the batch tests, obvious methane consumption was observed at similar rates of 368.2 and 406.7 μM day^–1^ in A1 and A2, respectively, when nitrate was served as the electron acceptor (Figure 1). The AOM rates were comparable to that of the parent bioreactor (301.1 μM day^–1^). In Stage II, after nitrate was depleted, methane consumption continued in A1 amended with Fe(III) citrate, although at a lower rate of 103.7 μM day^–1^ (Figure 1). However, no methane consumption was observed in the control group A2 supplied with sodium citrate (Figure 1). These results indicated that *Ca.* “*M. nitroreducens* TS” can sustain AOM by either reducing Fe(III) directly or by coupling to other potential Fe(III)-reducing bacteria *via* direct interspecies electron transfer.

### The Long-Term Bioreactor Performance With Ferrihydrite Amendment

In Stage I, methane and nitrate were simultaneously consumed (Figure 2 and Supplementary Figure 1), indicating that AOM was coupled to nitrate reduction. The average AOM rate was 308.6 μM day^–1^. Ammonium was also consumed along with nitrate (Supplementary Figure 1), which suggested that the anammox process occurred. In Stage II, a sharp decrease in the AOM rate was observed immediately after the replacement of nitrate with ferrihydrite (Figure 2). Though one magnitude lower than that in Stage I, methane was still consumed at a rate ranging from 51.7 to 63.1 μM day^–1^ before day 217, which indicated that AOM occurred in the presence of ferrihydrite. The AOM rate gradually dropped to 11.4 μM day^–1^ on day 464. From day 499 onwards, the AOM rate rebounded and reached 82.0 μM day^–1^ at the end of the incubation. The decrease in the AOM rate between day 551 and day 714 was due to the intensive withdrawal of biomass.

Dissolved Fe(II) production, along with methane consumption, suggested the reduction of ferrihydrite. A previous study has reported that dissolved Fe(II) only accounted for a small fraction (<5%) of the total Fe(II) produced (Cai et al., 2018), which may also be the case in this study. The concentration of dissolved Fe(II) rapidly increased and reached 2.1 mM on day 171, while a sharp drop was observed immediately after each addition of ferrihydrite. A decrease in dissolved Fe(II) was likely due to the absorption of dissolved Fe(II) onto the surface of ferrihydrite (Benjamin and Leckie, 1981; Schultz et al., 1987; Cai et al., 2018). In addition, the peak concentration of dissolved Fe(II) decreased along with the incubation, probably due to the accumulation of solid iron oxides.

At the beginning of Stage II, acetate was produced after nitrate was depleted, reaching 0.61 mM on day 72 (Figure 2), which was quickly consumed before day 115. Afterward, acetate could only be detected occasionally (<0.04 mM). In Stage II, nitrate was depleted, making the anammox process impossible. Biomass was likely degraded since ammonium was produced (Supplementary Figure 1). Anaerobic ammonium oxidation coupled to Fe(III) reduction (feammox) was reported previously (Huang and Jaffé, 2018). Despite the coexistence of ammonium and ferrihydrite for approximately 750 days, no ammonium consumption was observed (slight decrease in the ammonium concentration was attributed to the dilution by fresh medium) (Supplementary Figure 1). This result indicated that Fe(III) did not serve as an electron acceptor for ammonium oxidation, excluding the contribution of feammox to the production of dissolved Fe(II).

Taken together, continuing methane consumption in conjunction with significant dissolved Fe(II) production implies that AOM was driven by Fe(III) reduction during the long-term incubation.

### Shift in the Microbial Community During the Long-Term Incubation

**FIGURE 3:**
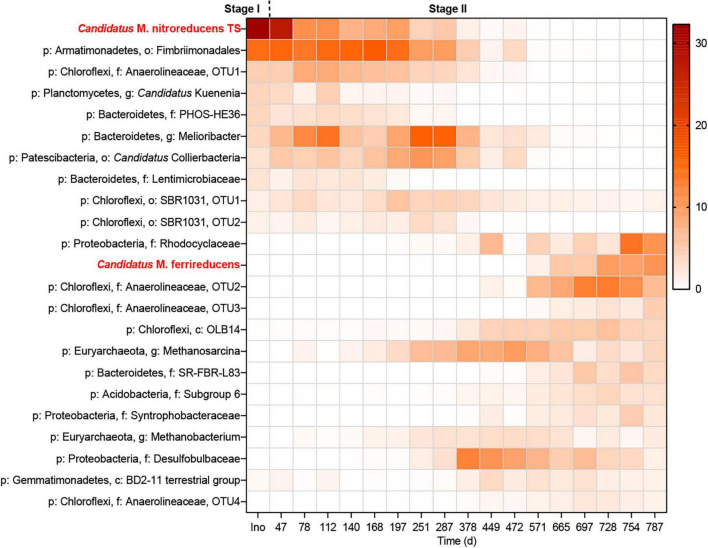
Heatmap showing the changes in the microbial community throughout the incubation period. The legend indicates the relative abundance.

**FIGURE 4:**
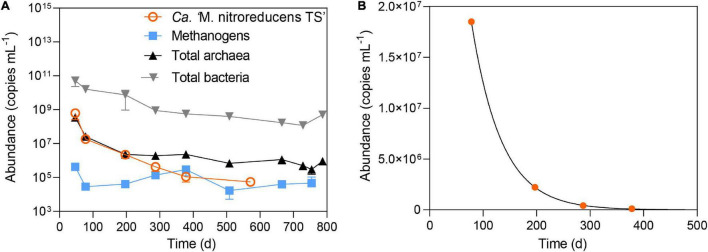
**(A)** The abundance of total archaea, total bacteria, methanogens, and *Ca.* “*M. nitroreducens* TS” throughout the incubation period. **(B)** Measured abundance of *Ca.* “*M. nitroreducens* TS” fitted with kinetic growth model.

The microbial community was monitored using 16S rRNA gene amplicon sequencing (Figure 3) and qPCR (Figure 4A). As shown in Figure 3, all the abundant organisms (>1%) in Stage I disappeared at the end of Stage II, while a new community was gradually formed. In Stage I, *Ca.* “*M. nitroreducens* TS” was highly abundant when nitrate was supplied (relative abundance of 28%), which was similar to that in the parent bioreactor (see the Supplementary Material). In Stage II, switching to ferrihydrite led to a decline in the total amount of microorganisms (Figure 4A) and production of ammonium, indicating biomass degradation. The total bacterial copy numbers dropped from 5.2 × 10^10^ (day 47) to 1.2 × 10^8^ (day 728) copies ml^–1^, and the total archaeal copy number decreased from 3.5 × 10^8^ (day 47) to 6.8 × 10^5^ (day 508) copies ml^–1^. In more than 200 days from the beginning of Stage II, *Ca.* “*M. nitroreducens* TS” was still abundant, though its relative abundance decreased to 6% on day 287. It further decreased to 0.1% on day 571. This was consistent with the qPCR results, which showed that the copy numbers of *Ca. M. nitroreducens* TS gradually decreased from 6.3 × 10^8^ (day 47) to 5.5 × 10^4^ copies ml^–1^ (day 571) (Figure 4A). In contrast, *Ca.* “*M. ferrireducens*,” another member of *Ca.* “Methanoperedenaceae” obligate for Fe(III)-dependent AOM (Cai et al., 2018), was detected on day 571. The population of *Ca.* “*M. ferrireducens*” further increased in the next 200 days and ultimately became abundant (11% on day 787). The copy numbers of total archaea were relatively stable when *Ca.* “*M. ferrireducens*” was increasing, which indicated that *Ca.* “*M. ferrireducens*” was enriched in the late period of Stage II. Overall, the abundance of *Ca.* “*M. nitroreducens* TS” or *Ca.* “*M. ferrireducens*” was generally in agreement with the AOM rate (Figure 2), indicating that they were responsible for methane consumption in different periods. The average cellular methane oxidation rate of *Ca.* “*M. nitroreducens* TS” before day 571 was 7 × 10^–16^ mol cell^–1^ day^–1^, which is comparable to those reported previously (4 × 10^–16^ to 5 × 10^–12^ mol cell^–1^ d^–1^) (Girguis et al., 2003; Raghoebarsing et al., 2006). Another known anaerobic methanotroph, *Candidatus “Methylomirabilis oxyfera*,” was initially detected at a very low level (0.5%) and was not detected from day 378. Thus, it unlikely contributed to methane consumption. Both 16S sequencing and qPCR results showed that acetotrophic methanogens were enriched before day 378 (Figures 3 and 4A). A batch test supplied with acetate apparently stimulated the growth of a methanogen close to *Methanosarcina mazei* and the production of methane (Supplementary Figure 2).

The relative abundance of *Ca. “K. stuttgartiensis*” declined from 4% to a low level (<0.1%). It indicated that the anammox bacterium was not functioning, which was also supported by the observation of a stable ammonium concentration in the bioreactor. Metal-reducing bacteria belonging to *Geobacter* were only occasionally detected at an exceedingly low level (<0.1%), suggesting that their contribution to Fe(III) reduction was negligible. In contrast, bacteria belonging to the family Rhodocyclaceae were enriched (11% on day 787). Isolation of a Fe(III)-reducing bacterium from this family has been reported (Cummings et al., 1999).

### Decay of *Ca*. “*Methanoperedens nitroreducens* TS” Under the Ferrihydrite Condition

To assess the decay rate of *Ca.* “*M. nitroreducens* TS,” qPCR readings between day 78 and day 380 were fitted using Equation 3 (Supplementary Material and Figure 4B). It resulted in a decay rate of 0.089 day^–1^. By comparing to that (*b* = 0.002 day^–1^) reported previously (He et al., 2013; Chen et al., 2014), the much higher decay rate suggested that the given iron condition was unfavorable to *Ca.* “*M. nitroreducens* TS”.

### Phylogenetic Placement of Key Microorganisms

**FIGURE 5:**
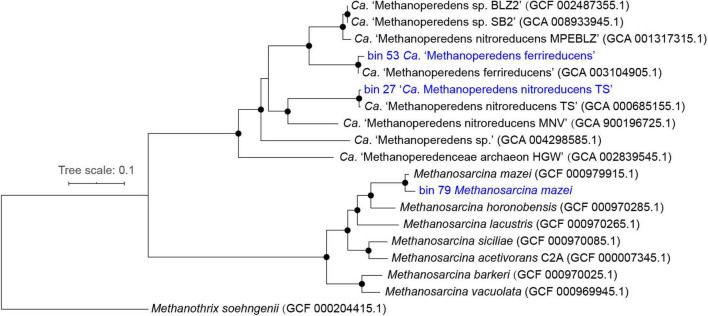
Rooted maximum-likelihood phylogenomic tree with draft genomes recovered in this study highlighted in blue. Bootstrap support between 95 and 100% were indicated as black circles.

Two metagenomic-assembled genomes (MAGs) of ANME archaea (bin_27 and bin_53; >95% completeness and <1% contamination) belonging to *Ca.* “Methanoperedenaceae” were recovered from the metagenomes. The genome tree showed that bin_27 formed a cluster with *Ca.* “*M. nitroreducens* TS” (Haroon et al., 2013) and bin_53 formed another cluster with *Ca.* “*M. ferrireducens*” (Cai et al., 2018; Figure 5). Further comparison of these two MAGs with their closest relatives (i.e., *Ca.* “*M. nitroreducens* TS” and *Ca.* “*M. ferrireducens*”, respectively) showed that the ANI of the two pairs were both close to 100% (Supplementary Figure 3). These results demonstrated that bin_27 and bin_53 were identical to *Ca.* “*M. nitroreducens* TS” and *Ca.* “*M. ferrireducens*,” respectively. One MAG (bin_79; >98% completeness and <4% contamination) belonging to methanogen was also retrieved from the metagenomes. The genome tree showed that bin_79 fell into a cluster with *M. mazei* (Youngblut et al., 2015) and shared an ANI of 98%.

### Multiheme *c*-Type Cytochromes and Acetate-Metabolizing Pathways in the Key Microorganisms

**FIGURE 6:**
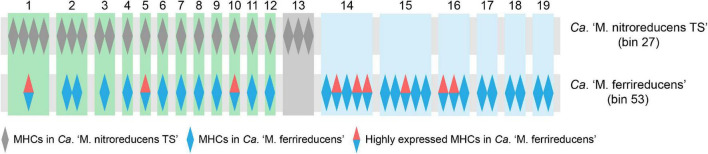
Orthogroups of multiheme *c*-type cytochromes (MHCs) from *Ca.* “*M. nitroreducens* TS” (bin_27) and *Ca.* “*M. ferrireducens*” (bin_53) based on orthology analysis. Each rectangle (green, gray, or light blue) is labeled by a number shown at the top and represents an orthogroup of homologous MHCs. The detailed information of the MHCs can be found in Supplementary Table 2.

*Ca.* “*M. nitroreducens* TS” (bin_27) and *Ca.* “*M. ferrireducens*” (bin_53) encoded 22 and 36 MHCs, respectively, which were hypothesized to facilitate EET (McGlynn et al., 2015; Cai et al., 2018; Leu et al., 2020). Orthology analysis showed that most of the putative extracellular MHCs from *Ca.* “*M. nitroreducens* TS” (bin_27) are orthologous to those from *Ca.* “*M. ferrireducens*” (bin_53), while 19 MHCs are exclusive to *Ca.* “*M. ferrireducens*” (Figure 6 and Supplementary Table 2). Moreover, 9 MHCs (LAFBCHPI_00375, 01883, 00984, 00699, 02494, 02368, 00697, 00966, and 00967) from *Ca.* “*M. ferrireducens*” (bin_53) exhibited high expression levels based on the metatranscriptomic data (Cai et al., 2018), which implied that these MHCs were involved in Fe(III) reduction. Notably, three of these highly expressed homologs (LAFBCHPI_00375, 01883, and 00984) were found in *Ca.* “*M. nitroreducens* TS” (bin_27) (Figure 6 and Supplementary Table 2). Metagenomic analysis also revealed that *M. mazei* (bin_79) possessed one MHC.

In addition, *Ca.* “*M. nitroreducens* TS” (bin_27) harbored two pathways, i.e., polyhydroxybutyrate (PHB) synthesis pathway and β-oxidation pathway (Eggers and Steinbüchel, 2013; Dibrova et al., 2014; Liu et al., 2016), for PHB metabolization. The PHB synthesis pathway was also found in *Ca.* “*M. ferrireducens*” (bin_53). *Ca.* “*M. nitroreducens* TS” (bin_27) and *Ca.* “*M. ferrireducens*” (bin_53) encoded acetyl-CoA synthetase (Acs/Acd), which is associated with acetate production. *M. mazei* (bin_79) was well-characterized as an acetotrophic methanogen (Westermann et al., 1989), and genes encoding acetate kinase (Ack), phosphate acetyltransferase (Pta), and acetyl-CoA synthase (Acs/Acd) were identified in its genome.

## Discussion

### Sustaining Fe(III)-Dependent Anaerobic Oxidation of Methane by *Ca*. “*Methanoperedens nitroreducens* TS” Over a Long-Term Period

This study showed immediate onset of AOM by the *Ca.* “*M. nitroreducens* TS”-abundant culture in the Fe(III) condition while no methane consumption without Fe(III) (Figure 1). These results supported that *Ca.* “*M. nitroreducens* TS” can carry out Fe(III)-dependent AOM in nitrate-depleted conditions. It is consistent with the observation of *Ca.* “*M. nitroreducens* MPEBLZ”, in spite of the large difference in the genomes between these two ANME species (ANI: 77.4%) (Arshad et al., 2015; Ettwig et al., 2016). Motivated by these results, a long-term incubation was conducted to evaluate the response of *Ca.* “*M. nitroreducens* TS” to Fe(III) amendment. Although the population kept decreasing after switching from nitrate to ferrihydrite, *Ca.* “*M. nitroreducens* TS” was the only known methane oxidizer for over 500 days of incubation. In the same period, AOM occurred concomitant with Fe(II) production, though the AOM rate dropped rapidly. These results suggested that *Ca.* “*M. nitroreducens* TS” sustain a long-term Fe(III)-dependent AOM.

Multiheme *c*-type cytochromes are ubiquitous in ANME-2 archaea and are hypothetically associated with Fe(III) reduction *via* EET (Haroon et al., 2013; Wang et al., 2014; McGlynn et al., 2015; Ettwig et al., 2016; Cai et al., 2018; Leu et al., 2020). *Ca.* “*M. nitroreducens* TS” (bin_27) encodes numerous MHCs, which may facilitate Fe(III) reduction. Until present, direct evidence that MHCs are involved in Fe(III) reduction in ANME archaea is still lacking. Metatranscriptomics analysis of several metal-reducing ANME archaea such as *Ca.* “*M. ferrireducens*” revealed that some specific MHCs were highly expressed during Fe(III) reduction (Cai et al., 2018; Leu et al., 2020). Comparison of *Ca.* “*M. nitroreducens* TS” (bin_27) and *Ca.* “*M. ferrireducens*” (bin_53) showed that most MHCs from *Ca.* “*M. nitroreducens* TS” are orthologous to those identified in *Ca.* “*M. ferrireducens*” (Figure 6). Intriguingly, three of the homologous MHCs were found to be highly expressed in *Ca.* “*M. ferrireducens*” (Cai et al., 2018). However, whether they are functionally active in *Ca.* “*M. nitroreducens* TS” still calls for future investigation.

### Population Changes in *Ca*. “*Methanoperedens nitroreducens* TS” and Other Microorganisms

Although *Ca.* “*M. nitroreducens* TS” drove Fe(III)-dependent AOM for a prolonged period, its population declined based on the results of 16S rRNA gene sequencing, qPCR, and kinetic analysis. The observation that this nitrate-reducing archaeon can reduce Fe(III) is similar to many sulfate-reducing microorganisms, which grow on sulfate but also can reduce Fe(III) (Jones et al., 1984; Coleman et al., 1993; Lovley, 1993). Those sulfate-reducing bacteria show no growth with Fe(III) as the sole electron acceptor (Lovley et al., 1993). They reduce Fe(III) probably for the following reasons: (1) *c*-type cytochromes as intermediate electron carriers may reduce Fe(III) inadvertently (Lovley et al., 1993); and (2) to facilitate Fe(III) depletion and create a favorable condition for sulfate reduction (Lovley, 2013). These reasons may also apply to *Ca.* “*M. nitroreducens* TS”. Nonetheless, it should be noted that high concentrations of dissolved Fe(II) and iron precipitates have accumulated in the bioreactor during incubation. The potential adverse effects of these compounds on *Ca.* “*M. nitroreducens* TS” need further investigation.

Acetate production was observed after switching from nitrate to ferrihydrite (Figure 2). PHB degradation from *Ca.* “*M. nitroreducens* TS”, which harbors two PHB-metabolizing pathways, may be one of the acetate sources. A previous study has demonstrated that acetate was produced by *Ca.* “*M. nitroreducens* TS” through limiting the supply of its preferable electron acceptor, i.e., nitrate (Cai et al., 2019). In this study, nitrate was completely removed from the bioreactor, probably creating an unfavorable condition for *Ca.* “*M. nitroreducens* TS”. Moreover, PHB is a carbon and energy source for many microorganisms (Roohi et al., 2018). Thus, it can be predicted that degradation of PHB may be a strategy for *Ca.* “*M. nitroreducens* TS” to cope with unfavorable conditions.

In addition, biomass was degraded throughout the incubation, particularly in the early- and mid-phase of Stage II (Figure 4A). Thus, biomass degradation may become another source of acetate and induce Fe(III) reduction by heterotrophic microorganisms. It was estimated that biomass degradation had the potential to contribute to approximately 11–31% Fe(II) formation between days 115 and 337. Bacteria belonging to the family Rhodocyclaceae were abundant in the new community (Figure 3). They are closely related to a denitrifier (*Denitratisoma* sp. strain TSA61) (Ishii et al., 2011); however, it was unlikely that they performed denitrification as no external nitrate or nitrite was fed into the bioreactor. Instead, a representative strain in this family, *Ferribacterium limneticum*, couples Fe(III) reduction to the oxidation of various organic acids such as acetate (Cummings et al., 1999). Therefore, it is hypothesized that these bacteria may use the products of cell lysis, including acetate, for Fe(III) reduction. Acetate may also be used by *M. mazei* for methanogenesis (Supplementary Figure 2).

### Environmental Implications

Environments characterized by abundant methane and Fe(III) are prevalent in marine (Beal et al., 2009; Riedinger et al., 2014; Egger et al., 2015) and freshwater systems (Crowe et al., 2011; Sivan et al., 2011; Norði et al., 2013). Growing studies have suggested that Fe(III)-dependent AOM is a ubiquitous process in those environments, where ANME archaea belongs to family *Ca.* “Methanoperedenaceae” inhabit. For example, the microorganisms were found in freshwater environments, including lake sediments (Weber et al., 2017; Su et al., 2020), riverbeds (Shen et al., 2019), cryotic thermokarst lakes (Winkel et al., 2019), and saline environments such as submarine permafrost undergoing salt diffusion (Winkel et al., 2018). Beyond that, related 16S rRNA gene sequences were also retrieved from marine settings (Schrenk et al., 2003; Reed et al., 2009). However, the link between Fe(III)-dependent AOM and these ANME archaea in the environment has escaped rigorous investigation (Beal et al., 2009; Crowe et al., 2011; Sivan et al., 2011; Amos et al., 2012; Wankel et al., 2012; Segarra et al., 2013; Riedinger et al., 2014; Egger et al., 2015; Egger et al., 2016; Weber et al., 2016; Egger et al., 2017).

This study shows that *Ca.* “*M. nitroreducens*” carries out Fe(III)-dependent AOM over a prolonged period, though the AOM rate dropped during the incubation. Coupling methane oxidation to Fe(III) reduction at a recognizable rate implies a potential role of *Ca.* “*M. nitroreducens*” in biogeochemical cycling of methane and iron. However, it is still puzzling that *Ca.* “*M. nitroreducens*” did not grow *via* Fe(III)-dependent AOM. Potential adverse conditions such as the toxicity of dissolved Fe(II) (Poulain and Newman, 2009) and overaccumulation of solid iron oxides may prevent the growth of *Ca.* “*M. nitroreducens*” and should be rigorously tested. In addition, *Ca.* “*M. nitroreducens*” was initially enriched *via* coupling AOM to nitrate reduction, showing the importance of nitrate in its growth (Haroon et al., 2013). Thus, how *Ca.* “*M. nitroreducens*” would respond to a co-feeding or interval feeding of nitrate and Fe(III) deserves further investigation.

Moreover, the capability of Fe(III) and nitrate reduction differentiates *Ca.* “*M. nitroreducens*” from *Ca.* “*M. ferrireducens*,” which lacks nitrate reductase and may use Fe(III) as its sole electron acceptor (Cai et al., 2018). This difference may have an impact on the distribution of these ANME species in the environment. For instance, nitrate fluctuation is common in many aquatic environments (Weber et al., 2020) and soils (Greenland, 1958), where methane and Fe(III) are also available. It can be speculated that the cooccurrence of Fe(III) and nitrate may create a niche accommodating both *Ca.* “*M. nitroreducens*” and *Ca.* “*M. ferrireducens*,” while *Ca.* “*M. nitroreducens*” may compete with *Ca.* “*M. ferrireducens*” for Fe(III) when nitrate is not available.

## Data Availability Statement

The datasets presented in this study can be found in the online repository: https://www.ncbi.nlm.nih.gov/bioproject/PRJNA611086 (BioProject accession PRJNA611086; MAG accession SRX8651446, SRX8651447, and SRX8651448).

## Author Contributions

CC, ZY, and SH conceived and designed the experiments. CC, JX, WL, and JP conducted the experiments. CC, GN, and YZ analyzed the data. CC, GN, YZ, XZ, BH, EM, ZY, and SH interpreted the data. CC and GN wrote the manuscript. All authors read, reviewed, and approved the submitted version.

## Conflict of Interest

The authors declare that the research was conducted in the absence of any commercial or financial relationships that could be construed as a potential conflict of interest.

## Publisher’s Note

All claims expressed in this article are solely those of the authors and do not necessarily represent those of their affiliated organizations, or those of the publisher, the editors and the reviewers. Any product that may be evaluated in this article, or claim that may be made by its manufacturer, is not guaranteed or endorsed by the publisher.
