# Supplementary material for: Response of the Anaerobic Methanotrophic Archaeon Candidatus “Methanoperedens nitroreducens” to the Long-Term Ferrihydrite Amendment
Source: Front Microbiol. 2022 Apr 18;13:799859. doi: 10.3389/fmicb.2022.799859 (PMC9058156; doi:10.3389/fmicb.2022.799859)
Supplement: Supplementary file 1 [file Data_Sheet_1.pdf]

Supplementary Material

Extended materials and methods

16S rRNA gene amplicon sequencing and analyses The extracted DNA was delivered to Australian Centre for Ecogenomics (ACE) at The University of Queensland for 16S amplicon sequencing. Universal primer set of 926F (5'-AAACTYAAAKGAATTGACGG-3') (Engelbrektson et al. 2010) and 1392wR (5'-ACGGGCGGTGWGTRC-3') was used to amplify V6-V8 regions of the 16S rRNA genes. Amplicons were sequenced by Illumina Miseq Platform and analyzed using the ACE pipeline (https://wiki.ecogenomic.org/doku.php?id=amplicon_pipeline_readme). This pipeline comprises a quality control module, amplicon clustering by QIIME2, and taxonomy assignment on representative OTU sequences through BLAST.

Quantitative real-time PCR The abundance of *Ca.* 'M. nitroreducens TS', methanogens, total bacteria, and total archaea were determined through quantitative real-time PCR (qPCR). The qPCR amplification was performed using a QuantStudio real-time PCR system (Thermo Fisher Scientific, U.S.) with the following mixture composition: 0.1 μL of each primer set (20 μM, Supplementary Table 1), 5 μL of SYBR Green PCR Master Mix (Thermo Fisher Scientific, U.S.), 2 μL of each DNA template (1ng μL^{-1}), and 3 μL of Milli-Q water. The qPCR program was an initial denaturation at 95 °C for 2 min, followed by 40 cycles of denaturing at 95 °C for 5 s, and annealing and extension at 60 °C for 20 s for *Ca.* 'M. nitroreducens', methanogens, and total archaea, or annealing at 55 °C for 20 s, and extension at 60 °C for 20 s for total bacteria. The standard curve was constructed from a series of 10-fold dilution of plasmid DNA, with R^2 values of at least 0.99 for all assays.

Evaluation of the contribution of biomass degradation to Fe(III) reduction To simplify the calculation, biomass degradation was evaluated after day 115 when acetate was consumed up. It was hypothesized that all the products from biomass degradation was used for Fe(III)

reduction and biomass degradation was correlated with ammonium production (Eq. 1). Ammonium production rate was calculated based on Supplementary Figure 1.

$$C_5H_7O_2N + 8H_2O + 20Fe^{3+} \rightarrow 20Fe^{2+} + 5CO_2 + NH_4^+ + 19H^+ \qquad (1)$$

Acetate amendment test A 100 mL of subsample was taken from the bioreactor in Stage II and transferred to a 180 mL bottle. Ar gas (100%) was used to flush the bottle to completely remove the residual methane. Acetate was added to the bottle to reach an initial concentration of 10 mM. Gas samples were taken three times each week for methane measurement. Liquid samples were taken every fortnight for acetate measurement. A biomass sample was taken on day 27 for 16S rRNA gene sequencing.

Kinetic evaluation of the growth rate of *Ca.* 'M. nitroreducens TS' The growth rate of *Ca.* 'M. nitroreducens' under ferrihydrite condition was evaluated based on the qPCR results (day 78378). According to a previous study (Chen et al. 2014), the net growth of *Ca.* 'M. nitroreducens' can be described as follows:

$$\frac{dX}{dt} = \left(\mu \frac{S_{CH4}}{S_{CH4}+K_{CH4}} \frac{S_{Fe}}{S_{Fe}+K_{Fe}} - b \frac{S_{Fe}}{S_{Fe}+K_{Fe}}\right) X \qquad (2)$$

Where X (copies mL^{-1}) is the copy number of *Ca.* 'M. nitroreducens'; μ (h^{-1}) is the maximum growth rate of *Ca.* 'M. nitroreducens'; b (h^{-1}) is the decay rate of *Ca.* 'M. nitroreducens'; S_{CH4} (mg CH_4 L^{-1}) is the concentration of methane; S_{Fe} (mg Fe L^{-1}) is the concentration of ferrihydrite; K_{CH4} (mg CH_4 L^{-1}) is the affinity constant of *Ca.* 'M. nitroreducens' for methane; K_{Fe} (mg Fe L^{-1}) is the affinity constant of *Ca.* 'M. nitroreducens' for Fe(III). In the long-term incubation, the average concentration of methane in the liquid phase was ca. 20 mg CH_4 L^{-1}. A methane affinity constant of 91 mg CH_4 L^{-1} for *Ca.* 'M. nitroreducens' was adopted although the real value is yet to be further determined (Lu et al. 2019). Though Fe(III) affinity constant for *Ca.* 'M. nitroreducens' is not available, it was reported that initial Fe(III) concentration of 2 mM adequately stimulated AOM in a linear course (Ettwig et al. 2016). In this study, the bioreactor was frequently replenished with ferrihydrite (initial concentration of >10 mM),

which was hypothesized to be sufficient for maintaining AOM by *Ca*. 'M. nitroreducens'. Thus, Eq. 2 can be simplified as:

$$\frac{dX}{dt} = (\mu' - b)X \qquad (3)$$

Where $\mu' = 0.18\mu$. Eq. 2 was implemented in AQUASIM 2.1d to estimate the value of the net growth rate for *Ca*. 'M. nitroreducens' based on the qPCR results.

DNA library preparation and metagenomic sequencing Paired-end (PE) libraries were prepared using the Nextera XT DNA library Preparation Kit (Illumina, U.S.) for DNA samples taken on day 168 and day 571, respectively. DNA libraries were sequenced on a NextSeq 500 (Illumina, U.S.) platform to obtain 2x150 bp reads with an average insert length of 300 bp. A total of 116,7 million and 89,9 million read pairs were generated for day 168 and 571, respectively.

Quality control, assembly and binning The quality control, assembly, binning, bin refinement and reassemble were carried out in a metagenomic wrapper tool MetaWRAP v1.2.1 with adjustments (Uritskiy et al. 2018). In brief, raw reads were trimmed based on adaptor content and PHRED scores (minimum 20) in Trim-galore v0.5.0 (https://www.bioinformatics.babraham.ac.uk/projects/trim_galore/). The two metagenomes were co-assembled using metaSPAdes v3.13.1 with kmers 21, 33 and 55 (Nurk et al. 2017). The total length of the co-assembly is 784 million bp, with N50 at 14,374. Three sets of MAGs were obtained using three binning strategies, i.e. MaxBin2 v2.2.4 (Wu et al. 2015), metaBAT2 v2.12.1 (Kang et al. 2019), and CONCOCT v1.0.12 (Alneberg et al. 2014), which were later consolidated into one superior set of MAGs that has maximum 10% contamination and are at least 70% complete, assessed by checkM v1.0.12 (Parks et al. 2015). Finally, MetaWRAP reassemble_bins module was utilized to improve N50, completeness and to reduce contamination.

Extended results

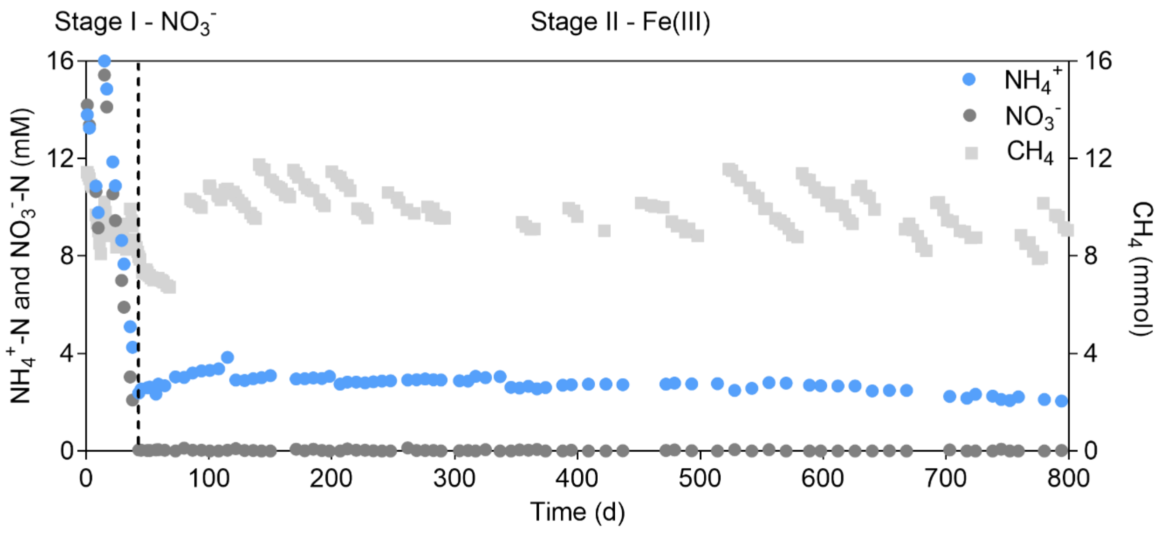

Supplementary Figure 1. Profiles of methane, nitrate and ammonium during the long-term incubation. Stage I and II were divided by the dashed line.

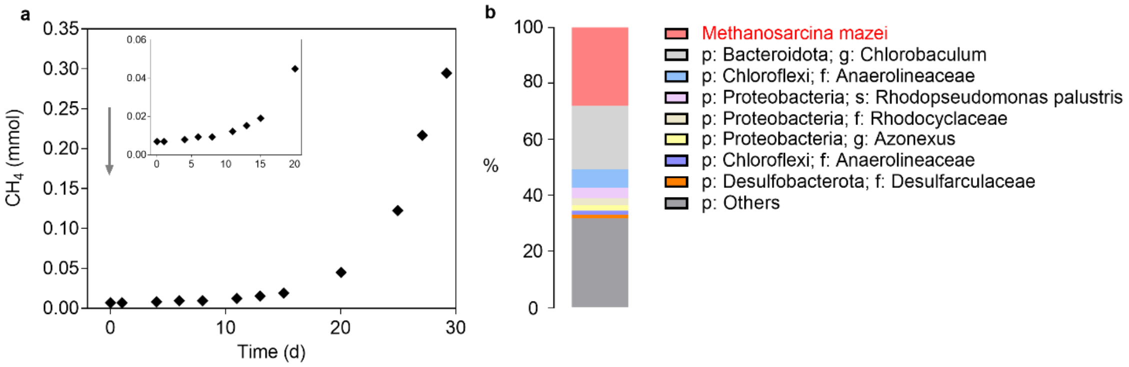

Supplementary Figure 2. Incubation of the subsample from the bioreactor with acetate amendment. **a** Methane production during the incubation; **b** Microbial community on day 27 of the incubation. Grey arrow indicates acetate addition.

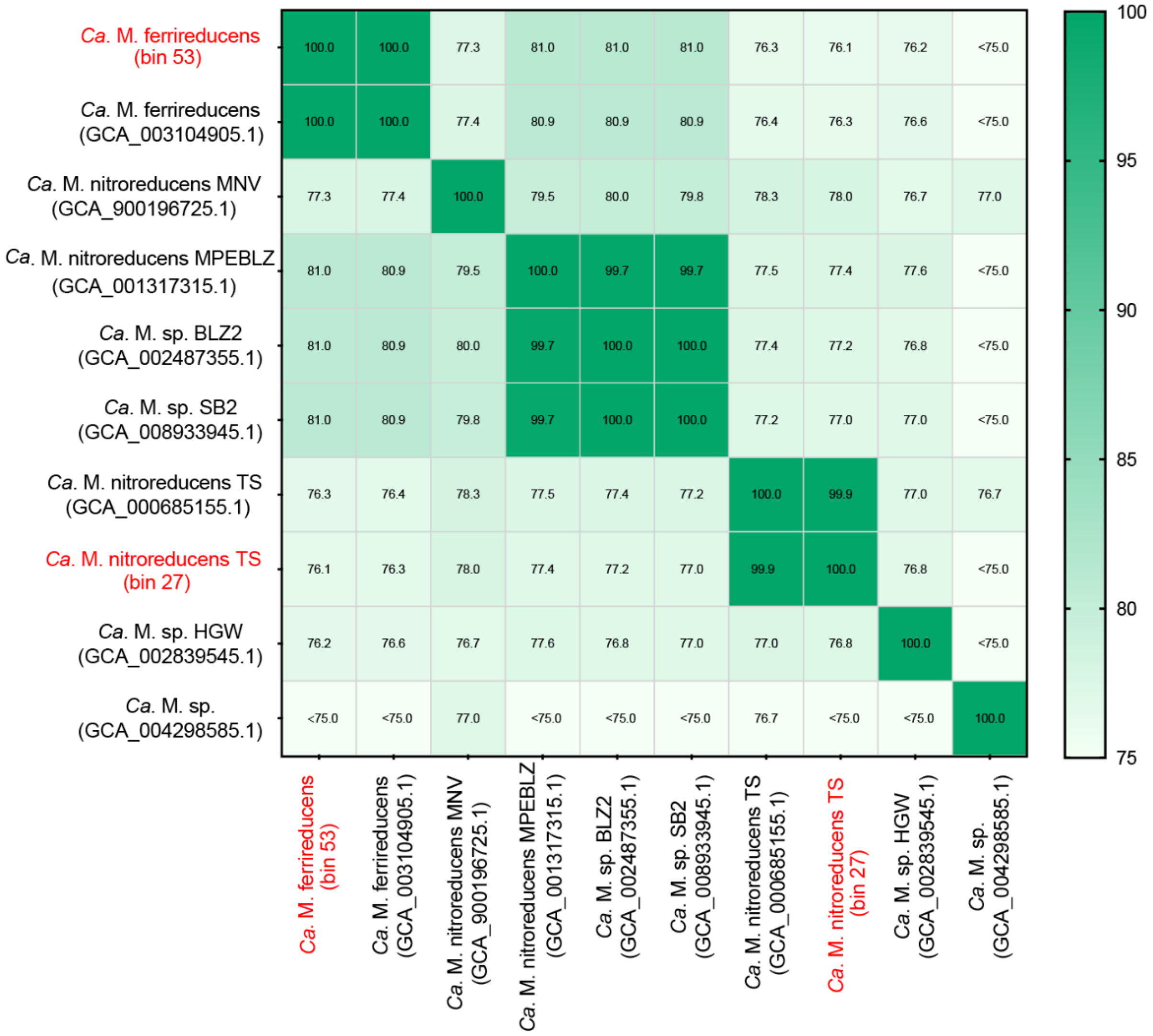

Supplementary Figure 3. Heatmap of average nucleotide identity (ANI) values of bins 27 and 53 with public available genomic sequences from the genus *Ca*. ‘Methanoperedens’.

Supplementary Table 1 Information of the primers used in qPCR amplification.

Primer name	Primer sequence 5’–3’	Targeting microorganism	Reference
McrA 159F	AAA GTG CGG AGC AGC AAT CAC C	*Ca.* ‘M. nitroreducens’	(Vaksmaa et al. 2017)
McrA 345R	TCG TCC CAT TCC TGC TGC ATT GC	*Ca.* ‘M. nitroreducens’	(Vaksmaa et al. 2017)
Met630F	GGA TTA GAT ACC CSG GTA GT	Methanogens	(Christophersen 2007)
Met803R	GTT GAR TCC AAT TAA ACC GCA	Methanogens	(Christophersen 2007)
341F	CCT ACG GGA GGC AGC AG	Total bacteria	(Muyzer et al. 1993)
515R	CCG CGG CTG CTG GCA C	Total bacteria	(Lane 1991)
344F	ACGGGGYGCAGCAGGCGCGA	Total archaea	(Casamayor et al. 2002)
915R	GTGCTCCCCCGCCAATTCCT	Total archaea	(Casamayor et al. 2002)

Supplementary Table 2. Orthology comparison of the putative extracellular MHCs in *Ca.* 'M. nitroreducens TS' (bin_27) and *Ca*. 'M. ferrireducens' (bin_53).

Orthogroup	bin_27	bin_53	Note
1	HOMMODLN_00616, 01338, 01392, 01476	LAFBCHPI_00984	Homologues in bin_27 and bin_53
2	HOMMODLN_00914, 00917, 00920	LAFBCHPI_02450, 02491	
3	HOMMODLN_01695, 02597	LAFBCHPI_01528	
4	HOMMODLN_00337	LAFBCHPI_00374	
5	HOMMODLN_00880	LAFBCHPI_01883	
6	HOMMODLN_01085	LAFBCHPI_01024	
7	HOMMODLN_01389	LAFBCHPI_00718	
8	HOMMODLN_01466	LAFBCHPI_01215	
9	HOMMODLN_01694	LAFBCHPI_02383	
10	HOMMODLN_02120	LAFBCHPI_00375	
11	HOMMODLN_02598	LAFBCHPI_01529	
12	HOMMODLN_02727	LAFBCHPI_02459	
13	HOMMODLN_01362, 01475, 02602		Only in bin_27
14		LAFBCHPI_00565, 00699, 00963, 00966, 00967	Only in bin_53
15		LAFBCHPI_00619, 02367, 02368, 02369, 02370	
16		LAFBCHPI_00697, 02494, 02496	
17		LAFBCHPI_00016, 01791	
18		LAFBCHPI_00706, 02449	
19		LAFBCHPI_02460, 02489	
20	HOMMODLN_02725		Not assigned
21		LAFBCHPI_00961	
22		LAFBCHPI_00964	
23		LAFBCHPI_01532	
24		LAFBCHPI_01833	

References

Alneberg, J., Bjarnason, B.S., de Bruijn, I., Schirmer, M., Quick, J., Ijaz, U.Z., Lahti, L., Loman, N.J., Andersson, A.F. and Quince, C. (2014) Binning metagenomic contigs by coverage and composition. Nature Methods 11(11), 1144-1146.

Casamayor, E.O., Massana, R., Benlloch, S., Øvreås, L., Díez, B., Goddard, V.J., Gasol, J.M., Joint, I., Rodríguez-Valera, F. and Pedrós-Alió, C. (2002) Changes in archaeal, bacterial and eukaryal assemblages along a salinity gradient by comparison of genetic fingerprinting methods in a multipond solar saltern. Environmental Microbiology 4(6), 338-348.

Chen, X., Guo, J., Shi, Y., Hu, S., Yuan, Z. and Ni, B.-J. (2014) Modeling of simultaneous anaerobic methane and ammonium oxidation in a membrane biofilm reactor. Environmental Science and Technology 48(16), 9540-9547.

Christophersen, C. (2007) Grain and artificial stimulation of the rumen change the abundance and diversity of methanogens and their association with ciliates, University of Western Australia.

Engelbrektson, A., Kunin, V., Wrighton, K.C., Zvenigorodsky, N., Chen, F., Ochman, H. and Hugenholtz, P. (2010) Experimental factors affecting PCR-based estimates of microbial species richness and evenness. ISME J 4(5), 642-647.

Ettwig, K.F., Zhu, B., Speth, D., Keltjens, J.T., Jetten, M.S. and Kartal, B. (2016) Archaea catalyze iron-dependent anaerobic oxidation of methane. Proceedings of the National Academy of Sciences 113(45), 12792-12796.

Kang, D.D., Li, F., Kirton, E., Thomas, A., Egan, R., An, H. and Wang, Z. (2019) MetaBAT 2: an adaptive binning algorithm for robust and efficient genome reconstruction from metagenome assemblies. PeerJ 7, e7359.

Lane, D. (1991) 16S/23S rRNA sequencing. Nucleic acid techniques in bacterial systematics, 115-175.

Lu, P., Liu, T., Ni, B.-J., Guo, J., Yuan, Z. and Hu, S. (2019) Growth kinetics of Candidatus 'Methanoperedens nitroreducens' enriched in a laboratory reactor. Science of the Total Environment 659, 442-450.

Muyzer, G., de Waal, E.C. and Uitterlinden, A.G. (1993) Profiling of complex microbial populations by denaturing gradient gel electrophoresis analysis of polymerase chain reaction-amplified genes coding for 16S rRNA. Applied and Environmental Microbiology 59(3), 695-700.

Nurk, S., Meleshko, D., Korobeynikov, A. and Pevzner, P.A. (2017) metaSPAdes: a new versatile metagenomic assembler. Genome Research 27(5), 824-834.

Parks, D.H., Imelfort, M., Skennerton, C.T., Hugenholtz, P. and Tyson, G.W. (2015) CheckM: assessing the quality of microbial genomes recovered from isolates, single cells, and metagenomes. Genome Research 25(7), 1043-1055.

Uritskiy, G.V., DiRuggiero, J. and Taylor, J. (2018) MetaWRAP—a flexible pipeline for genome-resolved metagenomic data analysis. Microbiome 6(1), 158.

Vaksmaa, A., Jetten, M.S.M., Ettwig, K.F. and Lüke, C. (2017) McrA primers for the detection and quantification of the anaerobic archaeal methanotroph 'Candidatus Methanoperedens nitroreducens'. Applied Microbiology and Biotechnology 101(4), 1631-1641.

Wu, Y.-W., Simmons, B.A. and Singer, S.W. (2015) MaxBin 2.0: an automated binning algorithm to recover genomes from multiple metagenomic datasets. Bioinformatics 32(4), 605-607.
